# Synthetic feature pairs dataset and siamese convolutional model for image matching

**DOI:** 10.1016/j.dib.2022.107965

**Published:** 2022-02-15

**Authors:** Houssam Halmaoui, Abdelkrim Haqiq

**Affiliations:** a ISMAC - Higher Institute of Audiovisual and Film Professions, Rabat, Morocco; b Hassan First University of Settat, Faculty of Sciences and Techniques, Computer, Networks, Mobility and Modeling laboratory: IR2M, Settat 26000, Morocco

**Keywords:** Learned features, Interest points, Keypoints, Matching model, Matching pipeline, Feature descriptors, Feature patch dataset, Feature pairs

## Abstract

In a previous publication [1], we created a dataset of feature patches for detection model training. In this paper, we use the same patches to create a new large synthetic dataset of feature pairs, similar and different, in order to perform, thanks to a siamese convolutional model, the description and matching of the detected features. We thus complete the entire matching pipeline. The accurate manual labeling of image features being very difficult because of their large number and the various associated parameters of position, scale and rotation, recent deep learning models use the result of handcrafted methods for training. Compared to existing datasets, ours avoids model training with false detections of the extraction of feature patches by other algorithms, or with inaccuracy errors of manual labeling. The other advantage of synthetic patches is that we can control their content (corners, edges, etc.), as well as their geometric and photometric parameters, and therefore we control the invariance of the model. The proposed datasets thus allow a new approach to train the different matching modules without using traditional methods. To our knowledge, these are the first feature datasets based on generated synthetic patches for image matching.

## Specifications Table

**Table:** 

Subject	Computer Vision and Pattern Recognition
Specific subject area	Image matching
Type of data	Image
How data were acquired	Datasets of feature patches, feature mosaic images and feature pairs were generated using Matlab and Python programming languages on an Intel i7 computer, then, used for model training with Python on GPU (Tesla P100-PCIE-16GB).
Data format	Raw Analyzed Filtered HDF5
Parameters for data collection	The images were carefully selected based on the different geometric and photometric variables of rotation, angle, luminance, contrast, blur and noise.
Description of data collection	The patch dataset consists of about 237K images 15×15, divided into two classes of 112K features and 125K non features. From these images, a dataset of about 2M pairs of features is created by considering the pairs of features with the same angle and contrast as similar patches (about 1M pairs), otherwise they are classified as different features (about 1M pairs).
Data source location	Institution: ISMAC - Higher Institute of Audiovisual and Film Professions City: Rabat Country: Morocco Latitude and longitude: 33.984494942490954, −6.865516338633437
Data accessibility	Repository name: Mendeley Data Data identification number: 10.17632/8jx8y9yfn5.2 Direct URL to data: https://data.mendeley.com/datasets/8jx8y9yfn5/2---
Related research article	H. Halmaoui, A. Haqiq, Convolutional sliding window based model and synthetic dataset for fast feature detection, in: The International Conference on Artificial Intelligence and Computer Vision, Springer, 2021, pp. 101-111. https://doi.org/10.1007/978-3-030-76346-6_10

## Value of the Data

• Recent image matching methods based on deep learning still use the result of traditional methods for training. This is due to the difficulty of manually and accurately labeling the position, orientation and scale of hundreds of features per image. The proposed synthetic datasets allow to avoid relying on handcrafted methods for the training of feature detection, description and matching models. Also, the use of the same patches for training the different models of the matching pipeline allows a better combination of these components.
• The datasets can be used by researchers working on one of the many applications of image matching, such as augmented reality or image registration, in order to train new models.
• The datasets are usable directly for training one or more modules of the matching pipeline. It is also possible to augment the dataset with new transformations, or to rely on the design methodology to create a new one, more specific to a certain application, for example by modifying the format and content of the images.
• The idea of using a synthetic dataset for image matching allows, by avoiding the use of traditional method results for training, and by being able to control accurately the geometric and photometric parameters of the features and generate them in very large numbers, opening up new possibilities for the training of matching models.
• The dataset simulates different geometric and photometric transformations in order to obtain matching models invariant to these transformations.

## 1 Data Description

We present in this article three datasets of synthetic images we have designed to train deep learning models for image matching. The first dataset, called patch dataset, is used to train convolutional and artificial feature detection models (first matching step). The second dataset, called mosaic images dataset, is used to train a convolutional sliding window model for fast feature detection. Finally, the third dataset, called feature pairs dataset, is used to train a siamese convolutional model for description and matching of the detected features (last two steps of the matching pipeline). Note that the first two datasets have already been the subject of a previous publication [1], but they will be presented here with more details about their designs, as well as some improvements that have been made. The main novelty here is the dataset of feature pairs and its use for training.

**Fig. 1:**
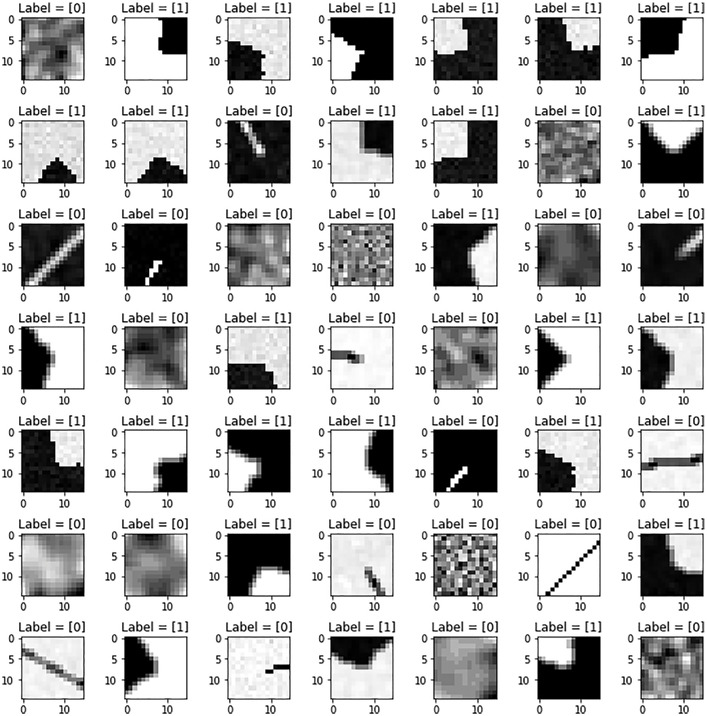
Random samples from the the patch dataset with different geometric and photometric transformations. Labels 1 and 0 correspond, respectively, to features (corners) and non-features (lines and uniform regions).

The Fig. 1 shows some random samples of the patch dataset, simulating different variations of angle, rotation, luminance, contrast, noise and blur. Fig. 3 shows an example of a mosaic image and the corresponding feature map that allows to locate the features. The Fig. 5 shows random samples from the feature pairs dataset.

**Fig. 2:**
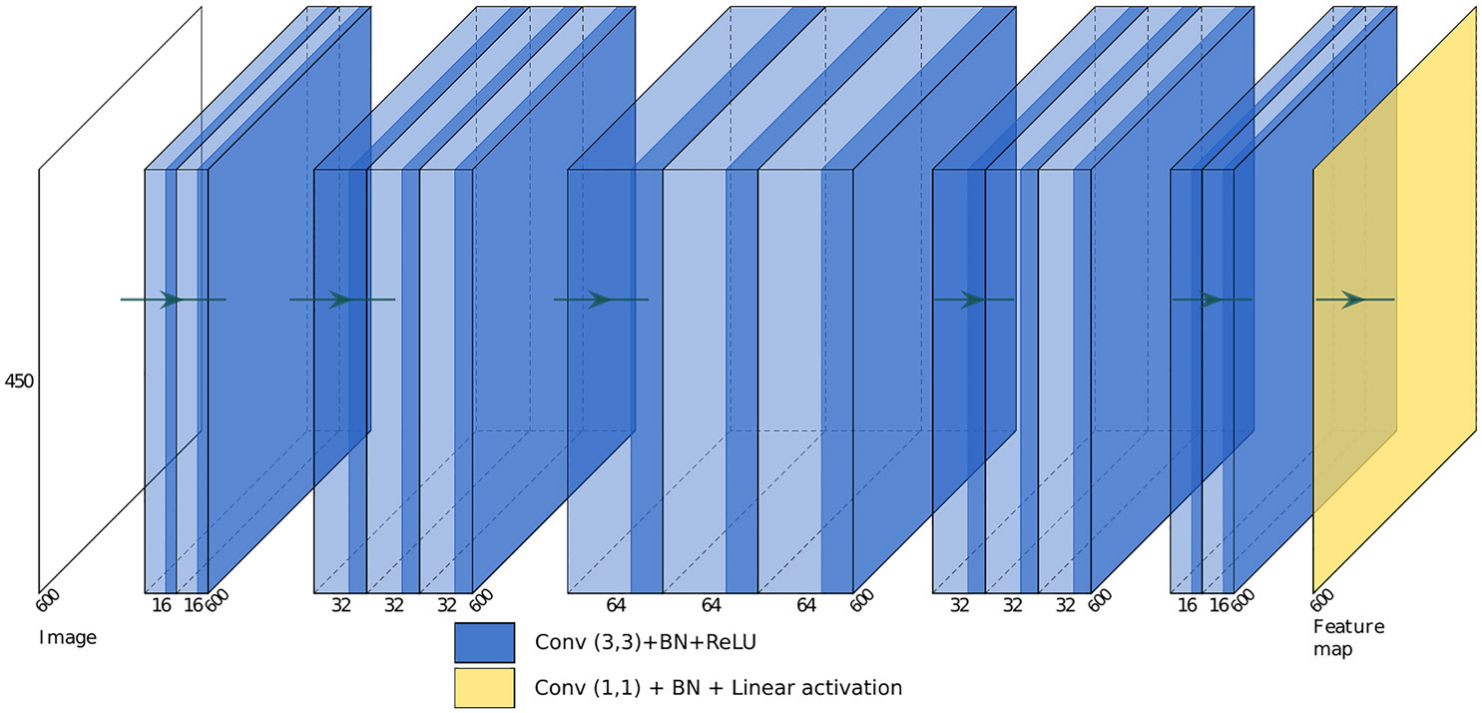
Convolutional sliding window model for fast feature detection.

**Fig. 3:**
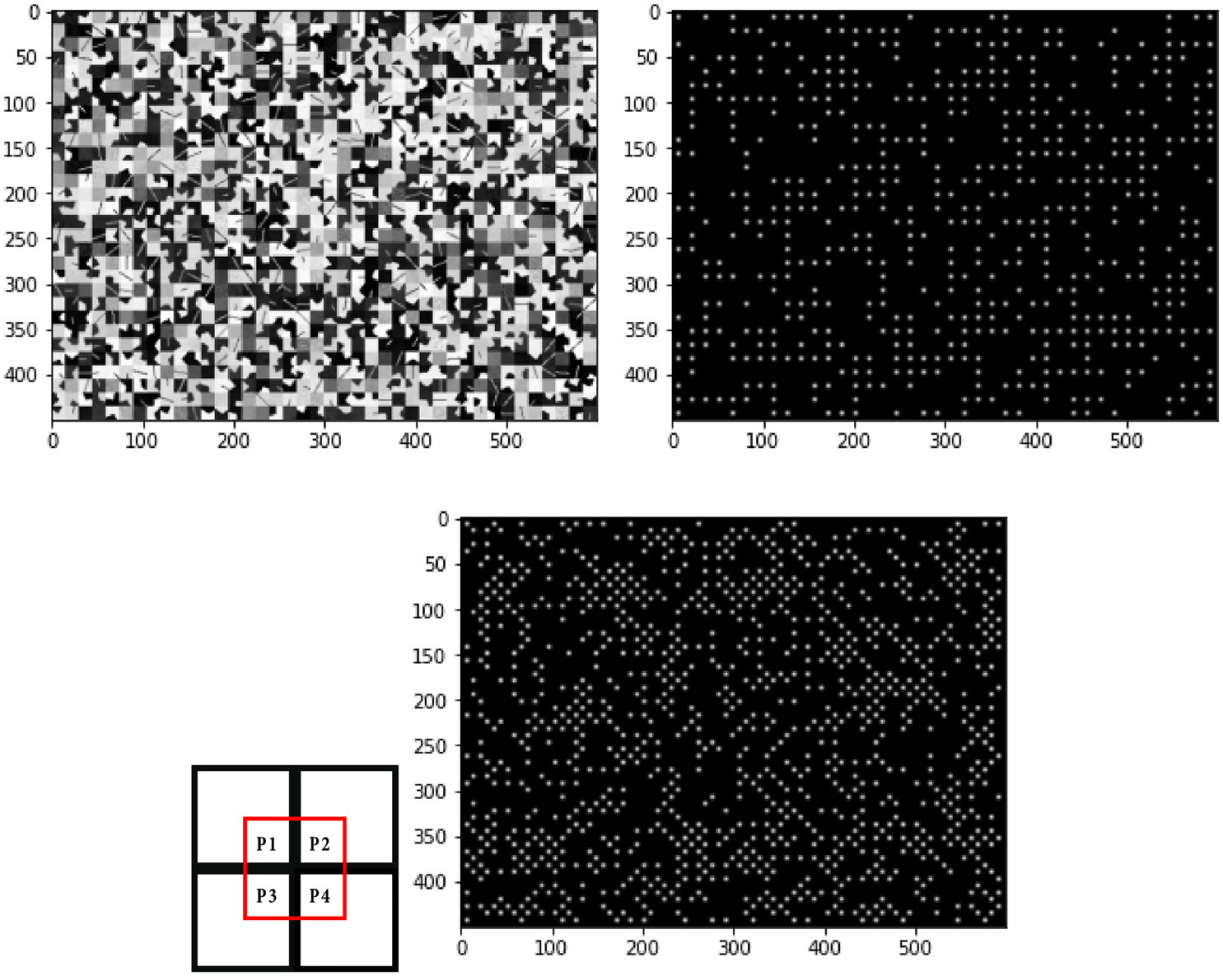
Example of a mosaic image (top left) and the corresponding feature map before (top right) and after adding the features at the intersection (bottom right). Illustration of the new features potentially generated at the intersection of four neighboring patches (bottom left).

**Fig. 4:**
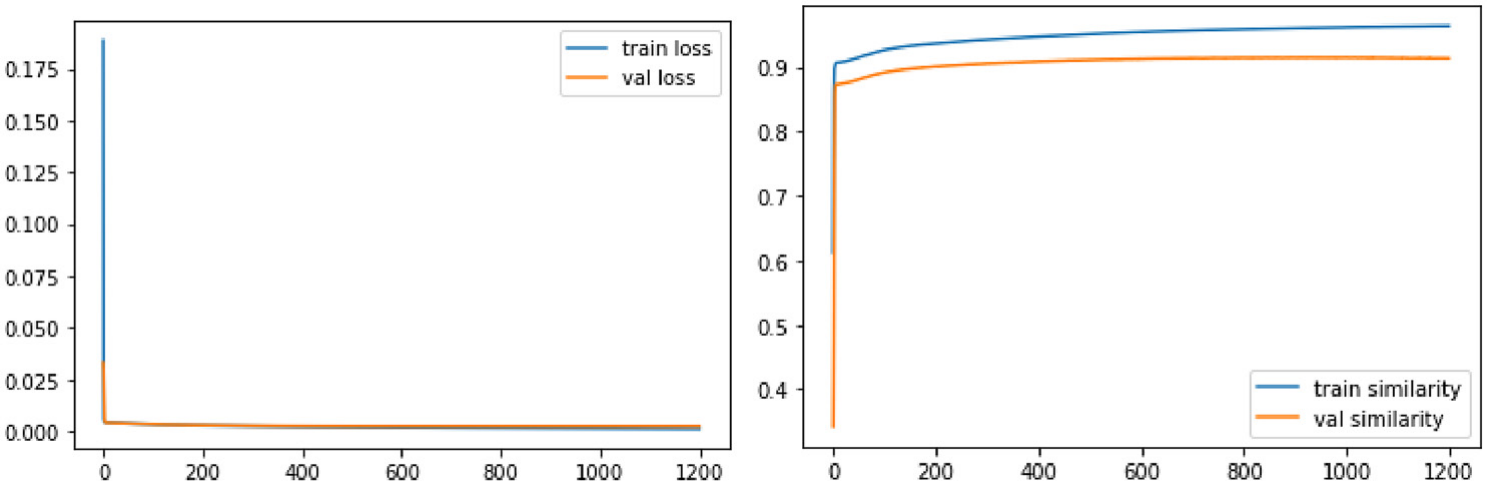
Training of the convolutional sliding window model on mosaic dataset: MSE Loss and cosine similarity metric, according to the number of iterations, for training set and validation set.

**Fig. 5:**
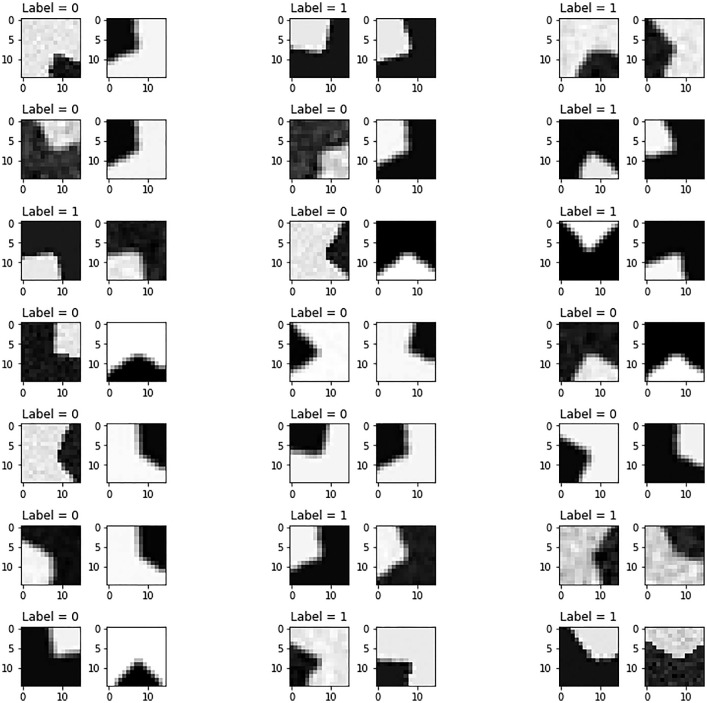
Random samples from the dataset of feature pairs. Similar pairs have a label 1 and different pairs have a label 0.

The different datasets are hosted in HDF5 format. The patch dataset is separated in two files of train set and test set in HDF5 formats. The mosaic dataset is separated into two files in HDF5 format of train and test sets. The feature pairs dataset is stored in HDF5 format in a single file.

Each dataset is accompanied by an IPYNB notebook code allowing to read and display the data as well as other information (format, type, number of samples) in order to facilitate its use.

The datasets are public and available to the community at [2].

## 2 Experimental Design, Materials and Methods

Feature matching consists in finding the correspondence between two images of the same scene acquired from different points of view. The applications in computer vision are numerous [3]: augmented reality, stereo, registration, camera pose, etc. Not all pixel regions of the image are reliable for matching. For example, lines and uniform regions are not locally unique in an image. Corners are more reliable features for matching [4]. The classical matching pipeline consists of 3 steps: feature detection, feature description and descriptor matching. Recent deep learning methods [5], [6], [7], [8], [9], [10], [11], [12], [13] use different models for each component. However, we found that these models still use feature datasets [14], [15], [16], [17], [18] based on the result of traditional handcrafted methods [19] for training. This is due to the difficulty to label in each image the position, orientation and scale of several hundreds of features accurately.

In fact, there are different types of datasets used to train these models. On the one hand, we have real image datasets [14], [15], [16] where features are localized by specific algorithms. On the other hand, the synthetic datasets use either real images to which synthetic geometric transformations are applied as in [17], [18], or synthetic images of scenes/objects where the feature patches are labeled manually or detected by other algorithms as in [20]. The novelty in the proposed datasets, is that we have synthesized feature patches, rather than images of scenes/objects. This avoids manual labeling of features or their detection by specific algorithms, and thus allows more localization accuracy, more control over the content, and avoids the algorithm’s false detection.

Based on this observation, in order to not rely on traditional methods, we have created in a previous article [1] a new dataset of synthetic features to allow the training of feature detection models, and which will be presented here in more detail, as well as some improvements that have been made.

In this paper, in order to complete the whole matching pipeline with only deep learning models, we propose a new large dataset of feature pairs, to train a siamese convolutional model of description and matching that predicts the similarity between pairs of features.

We will start by describing the feature patch dataset used for detection [1].

### 2.1 Patch dataset for feature detection

In this section, we will present the image patch dataset previously used and briefly described in [1]. In the following, we will provide all the details about its design.

The idea of creating a synthetic dataset is inspired by the traditional Harris detector, still widely used today, which classifies the image into three regions: corners, lines and uniform regions. We thus generated synthetic image patches of 15×15 dimensions for each of the three shapes, in order to train a CNN and ANN models [1], that classify the image regions into feature (corners) and non-feature (lines and uniform regions), as shown in Fig. 1.

For the corner features, we simulate, with constant steps: 13 corner angles varying from 90° to 130° (to avoid confusing them with edges and lines), 120 rotations from 0 to 360°, 18 contrasts greater than 0.6 with corners both darker and lighter than the background, a Gaussian noise $N(0,{\left(0.001\right)}^{2})$ (with and without), and a Gaussian blur with a kernel size 2×2 and a standard deviation σ=2/3 (with and without). We obtain a total number of features of 112320 (13×120×18×2×2).

For the line non-features, we simulated 90 rotations between 0 and 180° (symmetrical lines), 20 luminances and contrasts, 6 Gaussian noises with σ between 0 and 0.002, and 3 Gaussian blurs of kernel size between 1 and 3 with σ=1. For the half-line non-features, we simulated 180 rotations between 0 and 360×, 14 luminances and contrasts, 4 Gaussian noise with σ between 0 and 0.002, and 3 Gaussian blurs with kernel size between 1 and 3 with σ=1. For the uniform region non-features, we simulated 205 grey levels between 0 and 1, 34 Gaussian noises with σ between 0 and 0.01, and 9 Gaussian blurs of kernel size between 1 and 9 with σ=3. We obtain a total of 125370 non features.

The different values were chosen, empirically, after several tests, both to have a maximum of variabilities, visually perceptible corner and line structures, and to obtain balanced numbers of samples between the feature and non-feature classes.

The data were shuffled randomly before being stored in HDF5 format in two files of train (90%) and test set (10%).

The images are in gray level and coded on 8 bit (values between 0 and 255), it is thus necessary to normalize and convert them into float before training.

In [1], we used this dataset for training different ANN and CNN architectures, both shallow and deep, for feature detection.

For a faster detection with a convolutional sliding window model, we have created a feature mosaic dataset that we will present in the next section.

### 2.2 Mosaic dataset for fast feature detection

The dataset and the model that are presented in this section, are improved versions of those used in [1].

The convolutional sliding window model represented on Fig. 2 takes as input an image of dimensions 600×450 pixels and returns a feature map which allows to localize the features. It is a problem of localization by regression and not of classification as it is the case of the models using the first patch dataset. Note that the model presented here allows us to obtain a feature map that is denser than in its first version in [1]. Consequently, the dataset used is also slightly different than the one in [1].

To train this model, we have generated, from the patch dataset, mosaic images as well as the corresponding feature maps as shown in Fig. 3. The mosaic images are obtained by randomly concatenating the feature and non-feature patches in 600×450 images. Each image therefore contains 1200 patches (40×30 patches of dimensions 15×15). We obtain a total of 198 images from the 237690 patches. The remaining patches of the last image are filled with zeros. The feature maps are obtained by assigning to the features Gaussian shapes of size 15×15 and σ=15/9, normalized between 0 and 1. The center of the feature is therefore labeled with a value of 1 and its neighborhood with values less than 1 but not zero (because they also contain a part of the feature), this allows us to distinguish them from the non-featured patches to which we assign a value of 0. By concatenating the patches, we generate at their intersection, when one of them displays a significant contrast compared to all its neighbors, new corner features. We consider that the patch of dimension 15×15 located at the intersection of 4 patches is a feature, if one of the corners $P_{i}$ of the intersection (Fig. 3), satisfies the following condition:

(1) $$\left(Ctr\left(M_{i},M_{j}\right)\geq Th\right)\wedge \left(Ctr\left(M_{i},M_{k}\right)\geq Th\right)\wedge \left(Ctr\left(M_{i},M_{l}\right)\geq Th\right)$$

with Ctr(A,B) the contrast between two values A and B:

(2) $$Ctr\left(A,B\right)=\frac{max(A,B)-min(A,B)}{max(A,B)+min(A,B)}$$

$M_{i}$ is the median value of the corner $P_{i}$ of the intersection, with i∈{1,2,3,4}.

j,k and l are the indices of the neighbors $P_{j}$, $P_{k}$ and $P_{l}$ of $P_{i}$, with (i,j,k,l)∈{1,2,3,4}.

Th=0.6 is the contrast threshold used.

The mosaics and feature maps are stored in HDF5 format, in float type with values between 0 and 1, and separated in 179 images (90%) for the train set and 20 images (10%) for the test set.

We performed the model training with the following parameters : 80% train set; 10% validation set; 10% test set, epochs =1200; Adam Optimizer; decreasing learning rate between 0.01 and 0.00001; Mean squared error or MSE loss function; Cosine similarity metric.

The training took about 7 hours on GPU (Tesla P100-PCIE-16GB).

The Fig 4 shows the learning curves and the Table 1 shows the MSE and cosine similarity values at the end of the training.

**Table 1:** Values of MSE and cosine similarity after training the detection model on the mosaic dataset.

Loss (MSE)			Cosine Similarity		
Train	Validation	Test	Train	Validation	Test
0.0011	0.0022	0.0023	0.964	0.9137	0.9395

### 2.3 Feature pairs dataset for image matching

We present here the dataset of pairs of features, used for the training of a siamese convolutional model of feature description and matching. To build this dataset we use only the corner patches since the matching module has to find the correspondence between the detected features (corners). Note that we do not use all the corner patches because of the limits imposed by the RAM memory during the learning process as we will explain in more details later in this article. We retain only 15600 corners which allow us to generate the dataset of feature pairs. For this, we consider 13 corners as in the patch dataset, but only 10 contrasts and 30 rotations, 2 noise values, and 2 blur values.

We consider that two corners are similar if they have the same angle and the same contrast, but can have different rotations, noise and blur.

We thus build 130 subsets of similar features, each containing 120 features. Indeed, the number 120 corresponds to 30×2×2 (30 rotations, 2 noises and 2 blurs). The number 130 corresponds to 13×10 (13 angles and 10 contrasts).

We note $x_{j}^{i}$ the j-th patch of the subset i of similar patches, with i∈[1;130] and j∈[1;120].

To generate the feature pairs, we arrange the subsets $X^{i}$ of similar patches, with i∈[1;130], as follows:

(3) $$\left\{X^{1},X^{2},...,X^{130}\right\}=\left\{\left\{x_{1}^{1},...,x_{120}^{1}\right\},\left\{x_{1}^{2},...,x_{120}^{2}\right\},...,\left\{x_{1}^{130},...,x_{120}^{130}\right\}\right\}$$

From this data we generate a dataset of positive and negative feature pairs. We note P the set of positive pairs (similar patches) and N the set of negative pairs (different patches).

The set P is constituted by all the combinations of pairs in each subset $X^{i}$, including the pairs constituted by the same feature :

(4) $$P=\{\left(x_{m}^{k},x_{n}^{k}\right)|k\in \left[1;130\right],\left(m,n\right)\in \left[1;120\right]\}$$

Thus, we have a number of positive samples of :

(5) $$Card\left(P\right)=\left(130\times C_{120}^{2}\right)+\left(130\times 120\right)=943800$$

We use the algorithm 1 for the generation of the samples of the class P.

**Figure:**
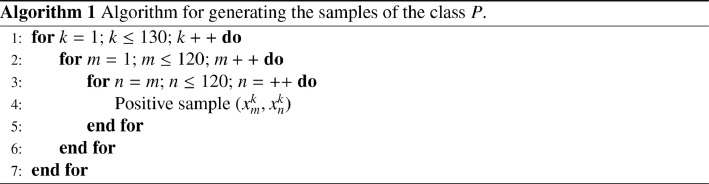

The set N of negative pairs corresponds to all the combinations of each feature of a subset $X^{i}$ with all the features of the other subsets $X^{j}$ with i≠j :

(6) $$N=\{\left(x_{p}^{m},x_{q}^{n}\right)|m\neq n,\left(m,n\right)\in \left[1;130\right],\left(p,q\right)\in \left[1;120\right]\}$$

So we have a number of negative samples of :

(7) $$Card\left(N\right)=C_{130\times 120}^{2}-\left(130\times C_{120}^{2}\right)=120744000$$

The very large number of negative pairs raises 2 problems. On the one hand, the two classes P and N are largely imbalanced. On the other hand, the amount of disk memory and especially RAM needed to store and process the set N is about 200Gio for 15×15 patch pairs coded on 32bits.

To solve these problems we consider the set $N^{*}$ of negative pairs that we obtain by taking one feature out of 3 of the subset $X^{i}$ and one feature out of 40 of the subset $X^{j}$. The steps 3 and 40 were chosen in order to obtain balanced P and $N^{*}$ classes. We used the algorithm 2 for the generation of the samples of the class $N^{*}$.

**Figure:**
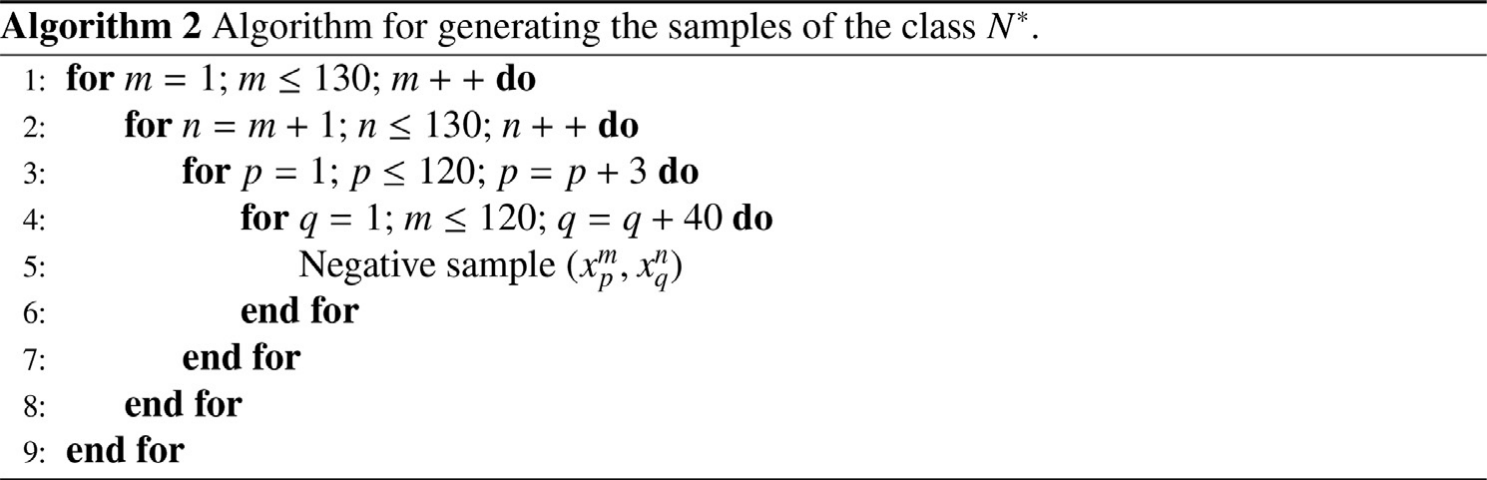

We obtain $Card(N^{*})=1006200$. The two classes P and $N^{*}$ are thus well balanced and the number of samples does not pose any problem of storage.

The total number of samples in the dataset is therefore 1.95M feature pair images. The Fig. 5 shows some random samples.

The samples are shuffled randomly and stored in HDF5 format.

**Fig. 6:**
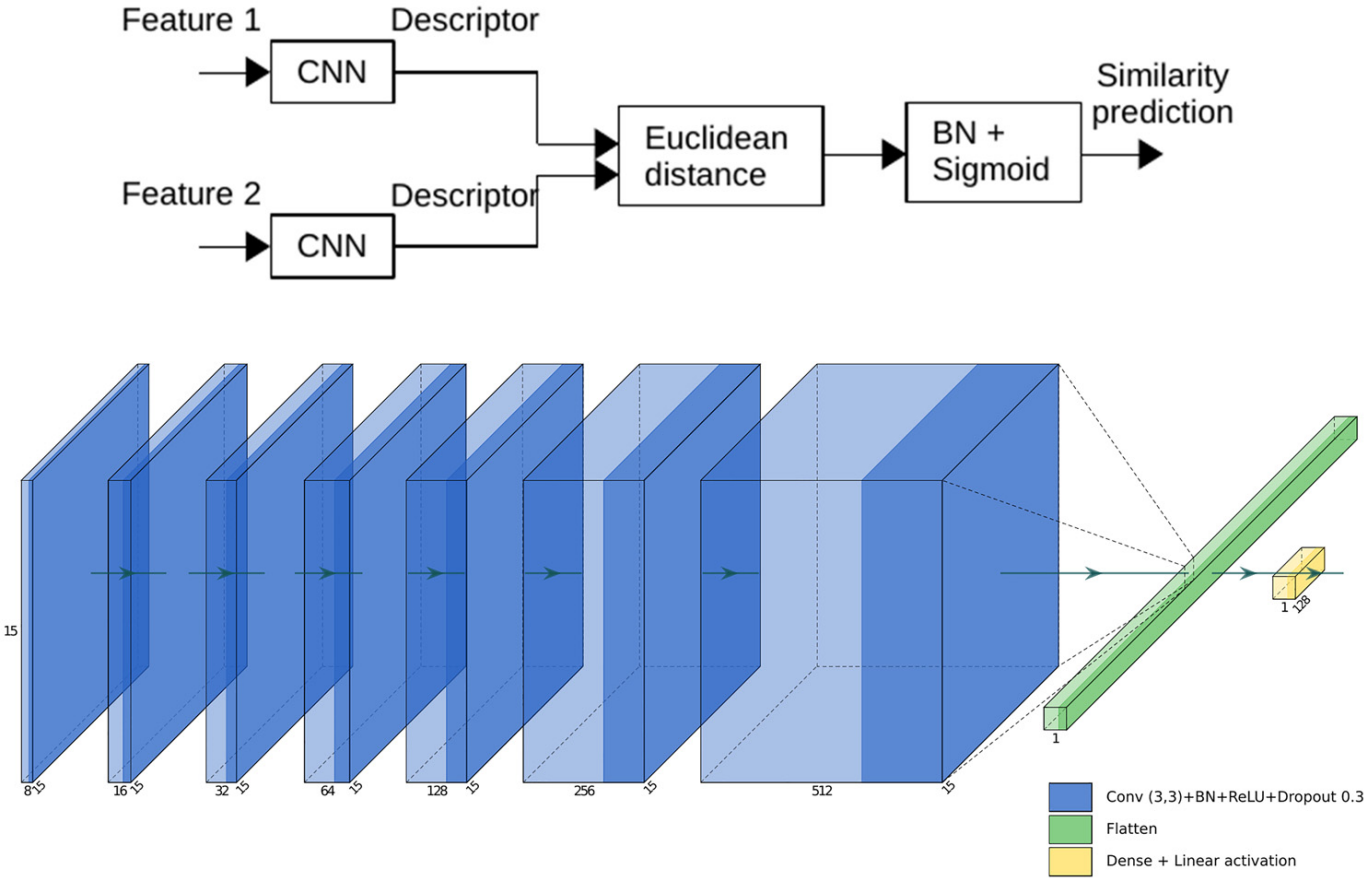
Siamese convolutional model (top) and CNN architecture used (bottom).

We used this dataset to train the siamese convolutional model represented on the Fig. 6.

The goal of the Siamese model is to differentiate patches by maximizing the Euclidean distance between the descriptors of pairs of different features:

(8) $$dist\left(a,b\right)={∥f\left(a\right)-f\left(b\right)∥}_{2}^{2}$$

with a and b two features, and f(a) and f(b) the corresponding descriptors obtained at the output of the CNN. For that purpose, we optimize the CNNs by using a contrastive loss function that maximizes the contrast (distance) between the positive and negative classes

(9) $$L=\left(1-Y_{true}\right)Y_{pred}^{2}+Y_{true}{\left(max\left(0,\alpha -Y_{pred}\right)\right)}^{2}$$

with alpha=1 the margin that ensures that the distance of the negative pairs is greater than the distance of the positive pairs.

**Fig. 7:**
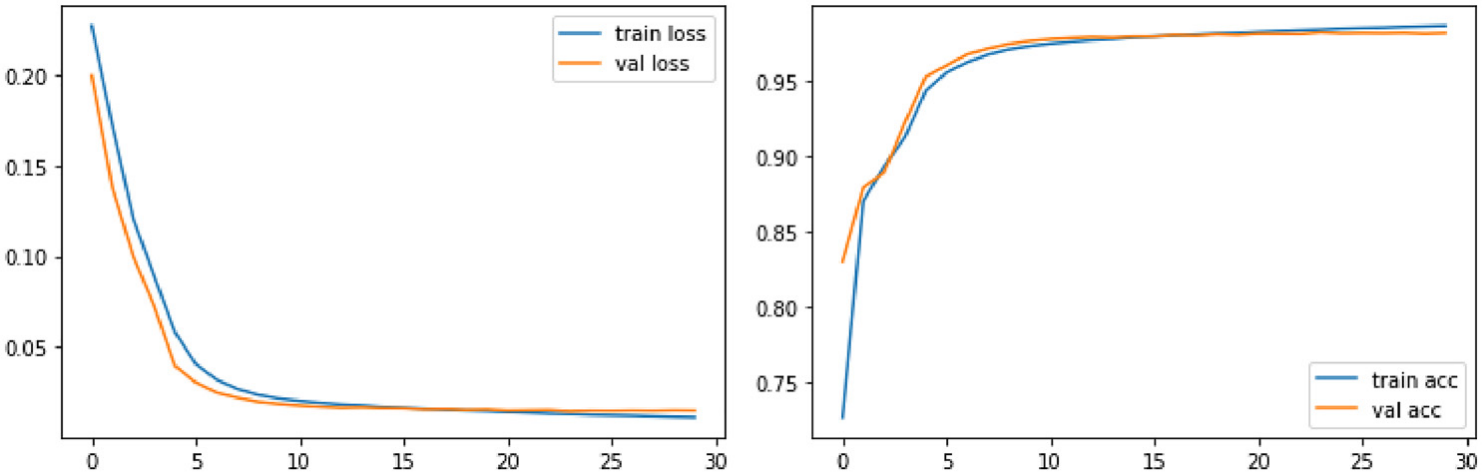
Training of the siamese model on feature pairs dataset: Loss and accuracy, according to the number of iterations, for training set and validation set.

**Fig. 8:**
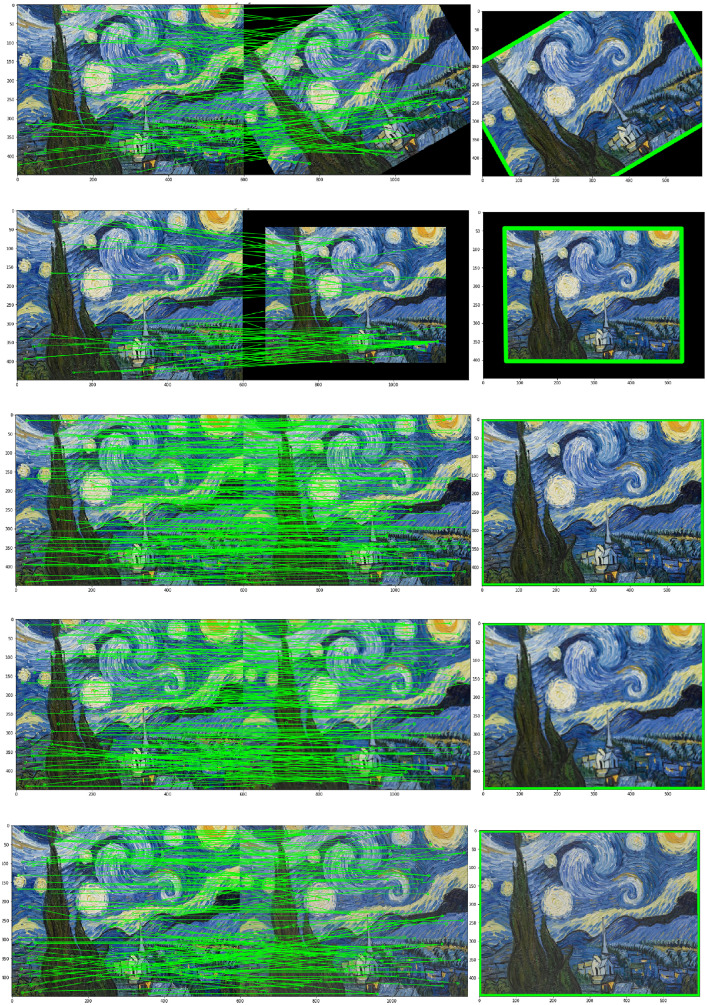
From top to bottom: Simulation of several transformations of 30× rotation, scale 0.8, noise $N(0,{\left(0.001\right)}^{2})$, motion blur with kernel 3×3 and linear change of luminance. Left: matching of the detected features. Right: estimation of the homography from the matched features.

$Y_{true}$ : The labels of the pairs.

$Y_{pred}$: The prediction of the model.

Note that both CNNs have the same architecture and learn the same parameters in order to compute the descriptors in the same way.

We used the following parameters for the training : 70% train set, 15% validation; 15% test set; batch size =512; epochs =30; decreasing learning rate between 0.1 and 0.0001; Adam Optimizer, Contrastive loss function; Accuracy metric.

The training took about 7 hours on GPU.

The Fig. 7 shows the learning curves and the table 2 the values of the contrastive loss and the accuracy at the end of the training.

**Table 2:** Contrastive loss and accuracy after learning the matching model on the feature pairs dataset.

Contrastive loss			Accuracy		
Train	Validation	Test	Train	Validation	Test
0.011	0.0146	0.0148	98.59%	98.13%	98.09%

### 2.4 Homography estimation

In order to test the combination of the two trained detection and matching models and how they generalize to new images, we applied different transformations to images and estimated the homography from the matched features. The matching outliers are eliminated with RANSAC. Fig. 8 shows some examples of estimation.

## CRediT authorship contribution statement

**Houssam Halmaoui:** Conceptualization, Methodology, Software, Formal analysis, Investigation, Data curation, Writing – original draft. **Abdelkrim Haqiq:** Supervision, Project administration, Writing – review & editing.

## Declaration of Competing Interest

The authors declare that they have no known competing financial interests or personal relationships which have, or could be perceived to have, influenced the work reported in this article.
